# Lack of imbalance between the master regulators *TTF1/NKX2-1* and *ΔNp63/p40* implies adverse prognosis in non-small cell lung cancer

**DOI:** 10.1038/s41598-024-52776-z

**Published:** 2024-01-30

**Authors:** Martina Vescio, Matteo Bulloni, Giuseppe Pelosi, Linda Pattini

**Affiliations:** 1 Department of Electronics, Information and Bioengineering, Politecnico di Milano, Piazza Leonardo da Vinci 32, 20133 Milan, Italy; 2 Present Address: CardioTech, IRCCS Centro Cardiologico Monzino, Milan, Italy; 3 Department of Oncology and Hemato-Oncology, University of Milan, Milan, Italy; 4 Inter-Hospital Pathology Division, IRCCS MultiMedica, Milan, Italy; 5 CardioTech, IRCCS Centro Cardiologico Monzino, Milan, Italy

**Keywords:** Cancer, Computational biology and bioinformatics, Systems biology, Oncology, Biomedical engineering

## Abstract

The transcription factors TTF1/NKX2-1 and ΔNp63/p40 are the counterposed molecular markers associated with the main Non-Small Cell Lung Cancer subtypes: TTF1 for adenocarcinoma, p40 for squamous cell carcinoma. Although they generally display a mutually exclusive expression, some exceptions exist simultaneously lacking or (very rarely) expressing both markers, either pattern being associated to poor prognosis. Hence, we quantitatively analyzed the relationship between their coordinated activity and prognosis. By analyzing the respective downstream transcriptional programs of the two genes, we defined a simple quantitative index summarizing the amount of mutual exclusivity between their activities, called Mean Absolute Activity (MAA). Systematic analysis of the MAA index in a dataset of 1018 NSCLC samples replicated on a validation dataset of 275 showed that the loss of imbalance between TTF-1 and p40 corresponds to a steady, progressive reduction in both overall and recurrence-free survival. Coherently, samples correspondent to more balanced activities were enriched for pathways related to increased malignancy and invasiveness. Importantly, multivariate analysis showed that the prognostic significance of the proposed index MAA is independent of other clinical variables including stage, sex, age and smoke exposure. These results hold irrespectively of tumor morphology across NSCLC subtypes, providing a unifying description of different expression patterns.

## Introduction

Lung adenocarcinoma (LUAD) and lung squamous cell carcinoma (LUSC) are the predominant subtypes of the heterogeneous category of non-small cell lung carcinomas (NSCLC), which accounts for 80% or more of all lung cancers^1^. TTF1/NKX2-1 (henceforth simply TTF1)^2^ and ΔNp63/p40^3,4^ (henceforth simply p40) are the most commonly used markers to classify NSCLC upon immunohistochemistry (IHC), with TTF1 confidently identifying LUAD and p40 LUSC^5–9^.

TTF1 is a transcription factor whose expression is highly biased in thyroid and lung tissues, where it exerts a crucial role in development and surfactant homeostasis. It is specifically expressed in distal airways, where it is a lineage marker for terminal respiratory unit cells. It plays an indispensable role in lung physiology, especially for regulation of surfactant protein pathways, and cannot therefore represent a therapeutic target per se^10^.

p40 refers to the N-terminally truncated isoforms of the transcription factor p63, a master regulator involved in a wide spectrum of functions encompassing cell fate determination, self-renewal, apoptosis and differentiation^11^. In this complex scenario, N-terminally truncated isoforms and full-length transactivating isoforms (TAp63) seem to regulate distinct molecular pathways^12^. In lung tissue, p40 is expressed in proximal airways: trachea, principal bronchus, bronchi and bronchioles, but not in the alveolus. Its expression is highly specific for LUSC, as opposed to TAp63, and due to its essential role in cell keratinization, it has become the most specific marker of LUSC^13^ according to the axiom “no p40, no squamous”^3,6–9^.

Both in physiology and pathology, the expression of TTF1 and p40 seems to be mutually exclusive^14^. However, about 15–20% of NSCLC lack both markers^15,16^ and feature poorly differentiated tumors with dismal prognosis^15–19^, similarly to the exceedingly rare instances of tumors with co-expression of both markers at the level of the same individual tumor cells^17–19^.

Here, we wanted to explore the transcriptional landscape depicted by the regulatory network of the master regulators TTF1 and p40 by relying on a large dataset of more than 1000 NSCLCs with clinical, pathologic and molecular annotations. To this aim, we applied a systems biology approach to estimate the activity of the two transcription factors in the specific context of NSCLC through the analysis of gene expression data available at The Cancer Genome Atlas (TCGA)^20,21^. We obtained a comprehensive picture of NSCLC transcriptional program downstream TTF1 and p40 that was relevant to prognosis, suggesting a more unifying description of existing subtypes, beyond tumor morphology.

## Results

### Identification of TTF1 and p40 regulatory networks

**Figure 1:**
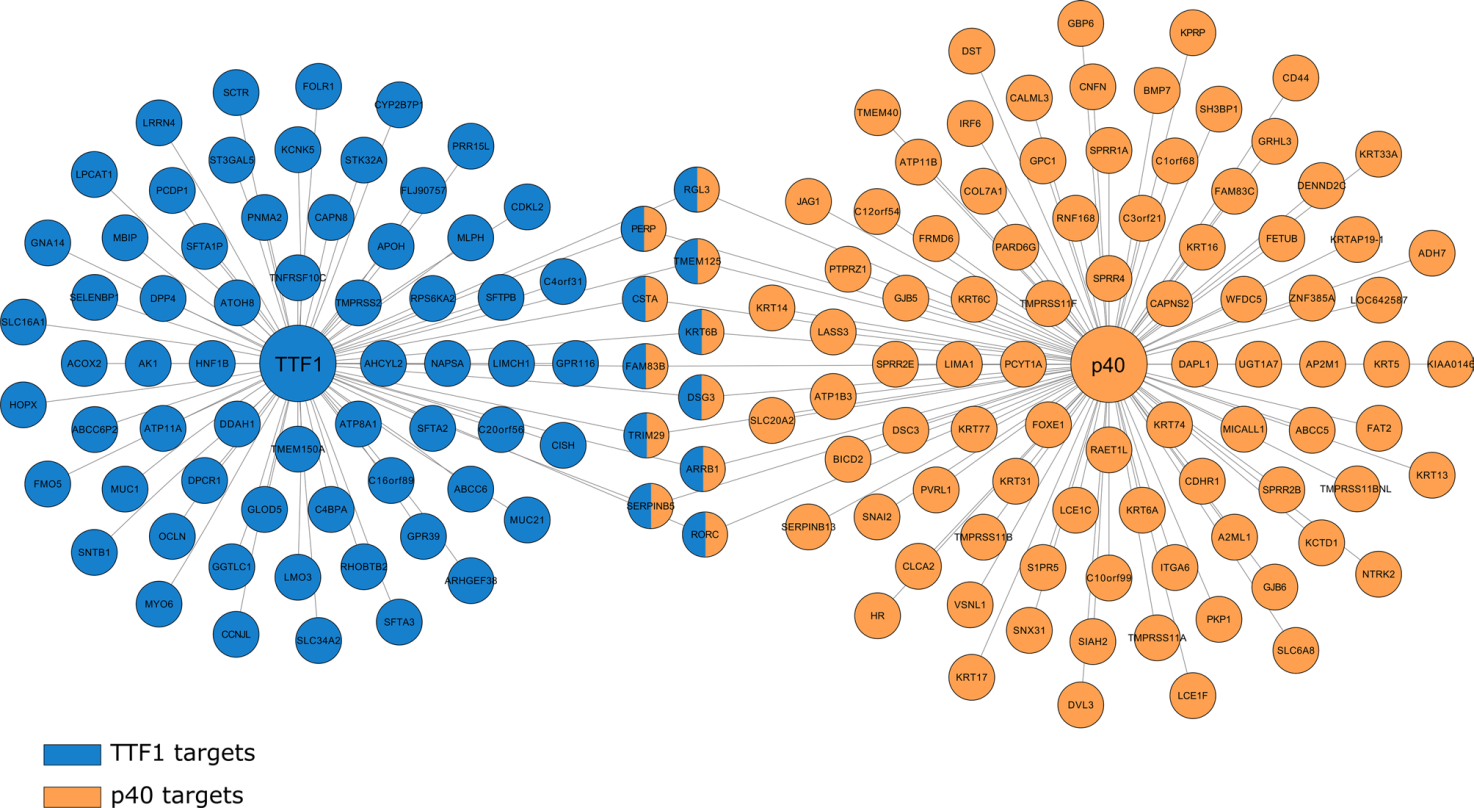
Gene co-expression network of TTF1 and p40 regulons. TTF1 network is represented in blue, p40 in orange. Targets shared by both transcription factors are shown as two-color nodes. Statistical dependence between targets and transcription factors expression levels was evaluated through mutual information. The associations reported present a significance of *p* < 10^–130^.

We aimed to determine the area of influence of TTF1 and p40 in the NSCLC transcriptional program. To do so, starting from gene expression profiling data, we identified a set of putative targets with the identification of most correlated genes for each of the two TFs across 1018 TCGA NSCLC samples (clinical characteristics of patients are reported in Supplementary Table S1). This procedure was first carried out separately for each gene isoform of the two TFs. For each TF, the aggregate regulon was then obtained as the union of the individual sets of targets of its isoforms, resulting in 135 genes for p40 and 111 for TTF1 (Fig. 1).

### Activity analysis confirms mutual exclusivity of TTF1 and p40

To evaluate to which extent TTF1 and p40 were driving the transcriptional regulation we estimated their respective activity value for each sample. This procedure provides a quantitative assessment of how effectively the TF influenced transcription in a sample by analyzing the expression values of its—direct and indirect—putative targets, i.e., the genes in the previously obtained regulons. A transcription factor is positively active (*activity* > *0*) if the genes for which it acts as an activator are expressed and those it represses are not expressed. A positive activity indicates therefore that the TF is successfully regulating the transcription in the cell. Conversely, a TF is negatively active (*activity* < *0*) in the opposite case, when the genes it should repress are expressed and those it should activate are not: the TF is not operating. As the activity value gets closer to zero the TF becomes neutrally active, thus indicating that it is acting on some targets, but is not able to fully drive the transcriptional regulation.

**Figure 2:**
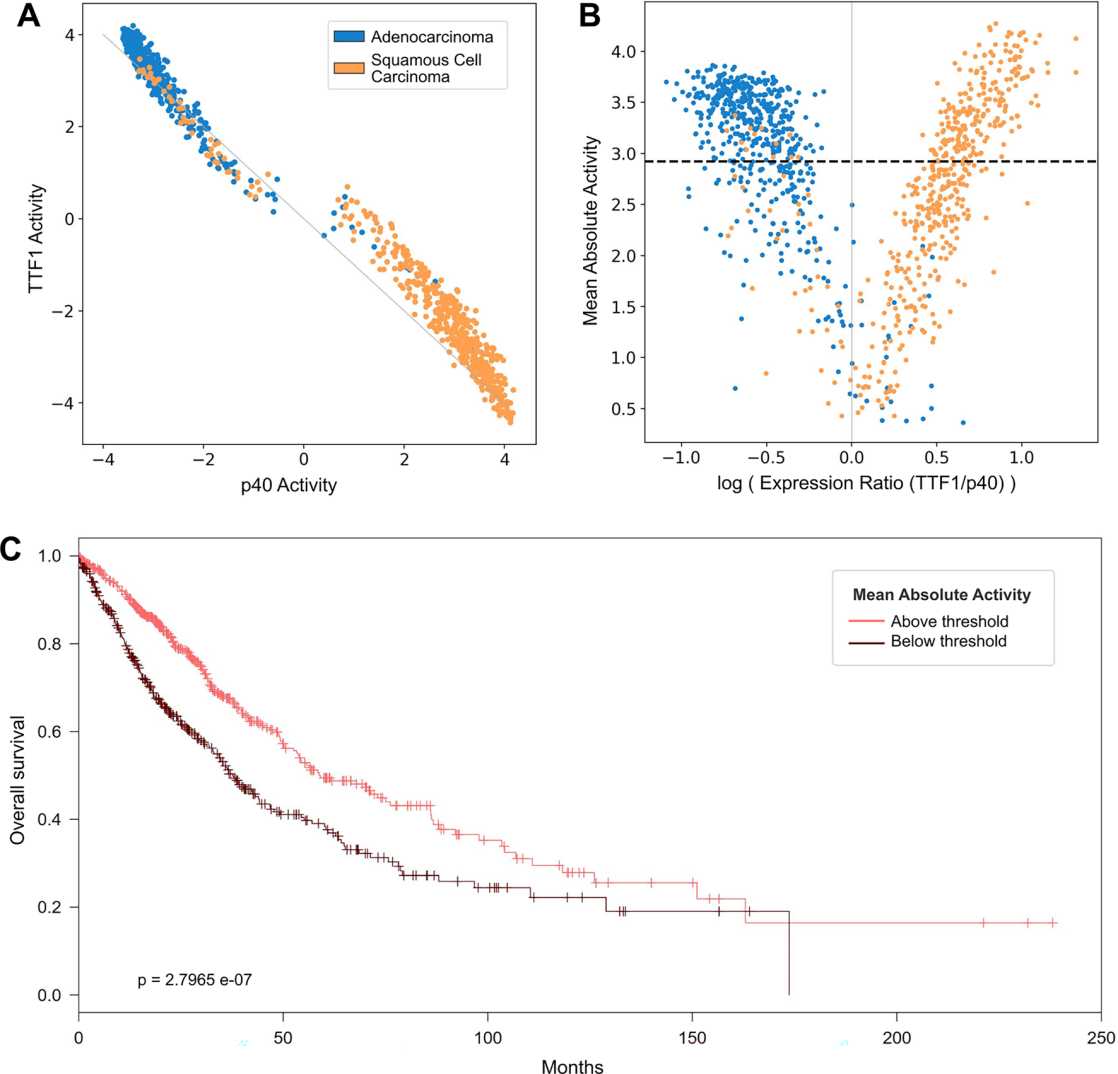
TTF1 and p40 activity analysis. (**A**) Relationship between the activity levels of TTF1 and p40 in each sample. The “switch-like” behavior of the two master regulators translates into a strong inverse linearity: as one shows a certain level of positive activity, its counterpart always shows the same level of negative activity. (**B**) Relationship between mean absolute activity (MAA) and the logarithm of the ratio between mRNA expression levels of TTF1 and p40. The MAA of a sample is defined as (| TTF1 activity | +| p40 activity |)/2, and tells how strongly either of the two regulators is prevailing on the other, i.e., how strongly one is positively active and the other negatively active. As the expression ratio of TTF1 and p40 gets close to 1 the MAA progressively decreases, meaning that the action of the two regulators balances out and neither is able to effectively drive differentiation. (**C**) Kaplan–Meier curve comparing overall survival rates of patients presenting a MAA above and below the optimal separation threshold (2.92) found through Cox regression, displayed in (b) as a dashed horizontal line.

Figure 2A shows the relationship between the two activity levels obtained for each sample. The plot discloses a clear inverse linear dependence between TTF1 and p40: as one shows a certain level of positive activity, the other prevalently shows the same level of negative activity. This provided further evidence that the two TFs were in fact competing for the control of the transcriptional regulation. The great majority of samples were characterized by a positive activity of the master regulator for their respective tumor subtype, and the most crowded regions of the scatterplot were those at the corners: a behavior “polarized” towards one end predominated, where either master regulator is active and the other one is switched off. On the contrary, progressively fewer samples appeared moving towards the center of the plot, where the activity levels of the two TFs start to balance out and neither of the two is able to prevail on the other.

A non-neglectable number of LUSC samples can be observed in the TTF1 positive / p40 negative region. Indeed, although LUAD is usually p40-negative, LUSC is reported to have about 38% TTF-1 positivity^4,22^.

### Mean absolute activity identifies samples with disrupted mutual exclusivity

To measure the predominance of one regulator over the counterpart we defined an index called *Mean Absolute Activity* (MAA), computed as semi-sum of absolute values of their activities:

$$MAA=\;\frac{{\left|{{\text{TTF}}1\;{\text{activity}}\left|+\right|{\text{p}}40\;{\text{activity}}}\right|}}{2}$$

High MAA values indicate that one regulator is clearly prevailing on the other and is thus individually driving the transcriptional regulation in the tumor sample. As the MAA decreases, the activities of the two TFs become comparable, leading to a mixed gene expression landscape. Figure 2B shows the MAA levels in our dataset, plotted against the ratio between the mRNA expression levels of TTF1 and p40 (more specifically, log_2_ (log_2_ (TTF1 expression) / log_2_ (p40-expression))). The plot highlights how the MAA progressively decreased as TTF1 and p40 reached a comparable level of expression, providing further evidence of competition and complementariness mechanisms between the two TFs: a switch-like relation that gets disrupted when neither regulator is able to overrule. Moreover, as the decrease in MAA is correlated with the ratio between the expression levels independently of their absolute values, the occurrence of this “broken switch” condition appeared to be triggered by an equilibrium of the two TFs at any level of expression.

### Lower MAA is associated with progressively worse prognosis

We evaluated Overall Survival (OS) and Recurrence-Free Survival (RFS) in patients with different MAA levels. We first compared samples with low and high values of MAA, dichotomized according to the threshold maximizing the difference in terms of survival (see Material and Methods). The threshold, displayed as a dashed horizontal line in Fig. 2B divided the patients in 584 with high MAA and 432 with low MAA. As displayed in Fig. 2C, patients with low MAA showed a significantly worse prognosis (*p* = 2.80 × 10^–07^). Similarly, low MAA patients exhibited significantly shorter recurrence-free survival times (*p* = 5.26 × 10^–03^, data not shown). These trends were confirmed when the analysis was carried out separately for LUAD and LUSC patients, thus suggesting that MAA assessment was independent of histologic subtyping.

**Figure 3:**
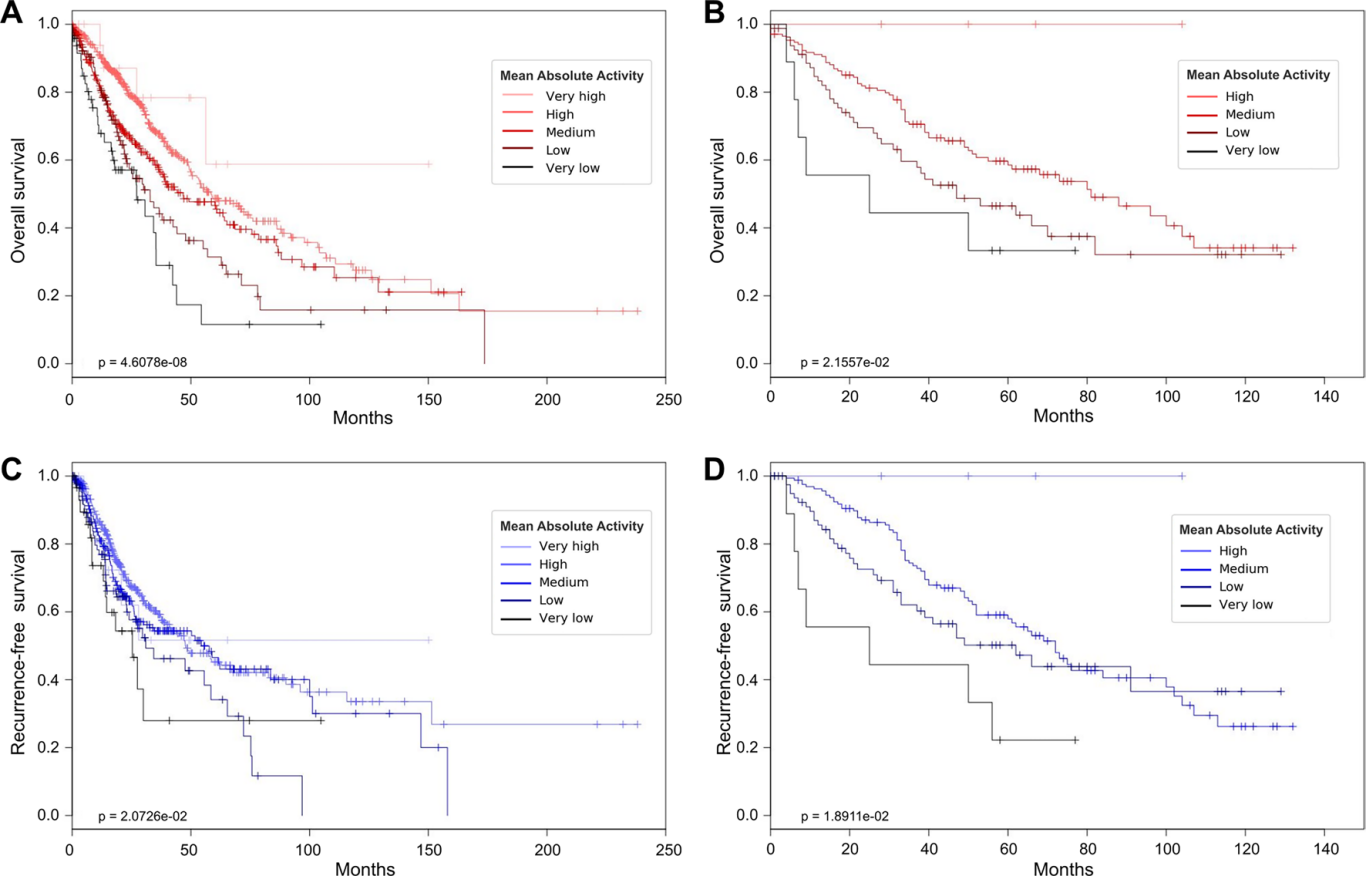
Kaplan–Meier curves comparing samples with different levels of mean absolute activity (MAA). (**A**–**D**) MAA intervals are defined as follows: very high: MAA > 4, high: 3 <  = MAA < 4, medium: 2 <  = MAA < 3, low: 1 <  = MAA < 2, very low: 0 <  = MAA < 1. (**A**, **B**) Overall survival in TCGA (**A**) and validation (**B**) datasets, respectively. (**C**, **D**) Recurremce-free survival in TCGA (**C**) and validation (**D**) datasets.

Then, to verify if there was a progressive reduction of the survival times as MAA decreased, we separated the patients in five groups according to their level of MAA. We found that, for both OS and RFS, the shortening of the survival times was indeed progressive, following the decrease of MAA. This pattern was particularly evident for overall survival (OS: *p* = 4.61 × 10^–08^, RFS: *p* = 0.03; Fig. 3A,C).

Ultimately, the results obtained highlighted that the gradual loss of mutual exclusivity between TTF1 and p40 was correlated with an equally progressive worsening of the prognosis and a greater risk of recurrence, irrespectively of the tumor subtype.

Notably, a Cox multivariate model of OS indicated that the prognostic performance of MAA is independent of other clinical variables including stage, sex, age and smoking history (assessed as pack-years). Univariate analysis for each stage category was also performed, confirming MAA prognostic significance for stage I, II and IIIA (see Table 1).

**Table 1:** Multivariate and univariate survival analysis of MAA in the TCGA dataset.

Predictor	HR (95% CI)	*p*-value
Multivariate		
MAA	0.74 (0.65–0.84)	3.73E−06
Sex	1.26 (0.98–1.62)	0.0729
Stage II	1.40 (1.06–1.84)	0.0174
Stage III	1.82 (1.35–2.46)	8.17E−05
Stage IV	2.76 (1.60–4.75)	0.0003
Age	1.02 (1.00–1.03)	0.0132
Smoking (pack year)	1.00 (1.00–1.00)	0.7684
Univariate		
MAA (Stage I)	0.78 (0.66–0.92)	0.0034
MAA (Stage II)	0.68 (0.55–0.83)	0.0002
MAA (Stage IIIA)	0.66 (0.50–0.87)	0.0037
MAA (Stage IIIB)	0.58 (0.32–1.05)	0.0733
MAA (Stage IV)	0.67 (0.45–1.01)	0.0559

Prediction significance of MAA adjusted for clinical variables in the TCGA dataset and its univariate analysis within each stage category. MAA, Mean Absolute Activity; HR, hazard ratio; CI, confidence interval.

### Prognosis predictivity of MAA is confirmed in an independent dataset

To validate the results obtained, we employed gene expression data from an independent dataset (NCBI GEO GSE41271) containing 275 NSCLC samples (clinical characteristics of patients are reported in Supplementary Table S2), where expression profiles were available at gene level (without distinction of alternative transcripts). Despite this approximation, the analysis remarkably confirmed the results obtained on the TCGA dataset: patients exhibited progressively worse prognosis as MAA decreased (Fig.3B,D).

### Annotation analysis confirms prognostic significance and activation of pathways related to invasiveness in low MAA samples

**Figure 4:**
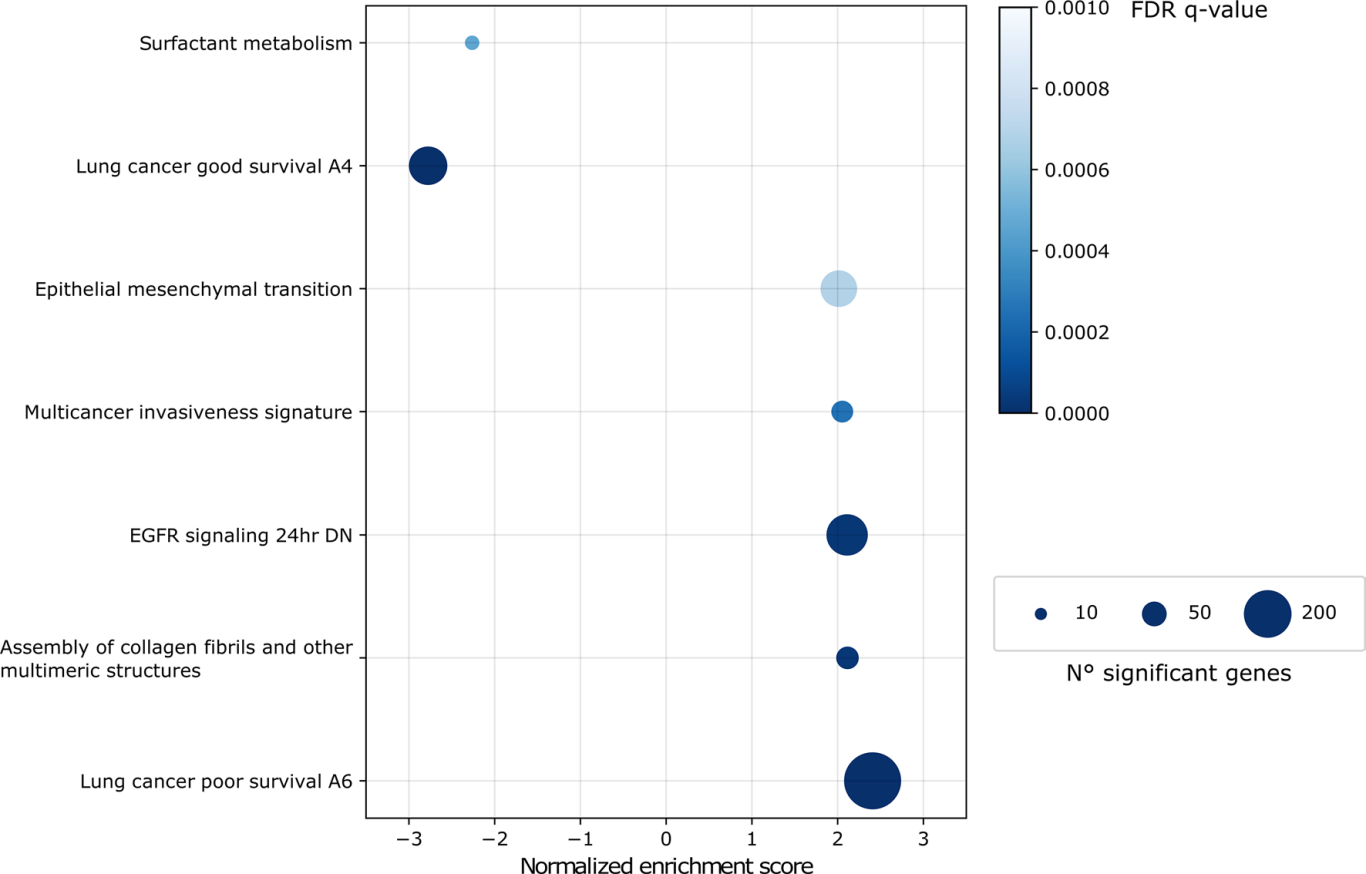
Significantly enriched gene sets in the comparison between low MAA and high MAA tumors. The colorbar represents significance expressed as FDR q-value and the circle dimension is proportional to the number of genes annotated with a specific term.

Functional annotation analysis was performed to compare gene expression in low versus high MAA samples. A selection of the most significant results, confirmed in the validation dataset, is shown in Fig. 4 (see Supplementary Table S3 for the complete list). Already characterized gene signatures for lung cancer survival^23^ were coherently significant (good survival gene set was under-expressed and poor survival gene set was over-expressed in low MAA samples) along with a *multicancer invasiveness* signature^24^. Samples with low MAA showed enrichment for *epithelial-mesenchymal transition (EMT)* and *collagen formation*, pertaining to extracellular matrix organization as usually observed in more aggressive tumors. Also, low MAA tumors showed a significant downregulation of *surfactant metabolism*.

Finally, we evaluated the correlation between MAA and the expression values of all genes to identify the one that most closely resembled the MAA behavior: it resulted to be the circadian gene hepatic leukemia factor (HLF), a transcription factor member of the proline and acidic-rich (PAR) protein family (Spearman’s r: 0.44, FDR = 4.75 × 10–46), whose prognostic value has been recently reported^25^.

### Low MAA tumors show enhancement in EMT markers

**Figure 5:**
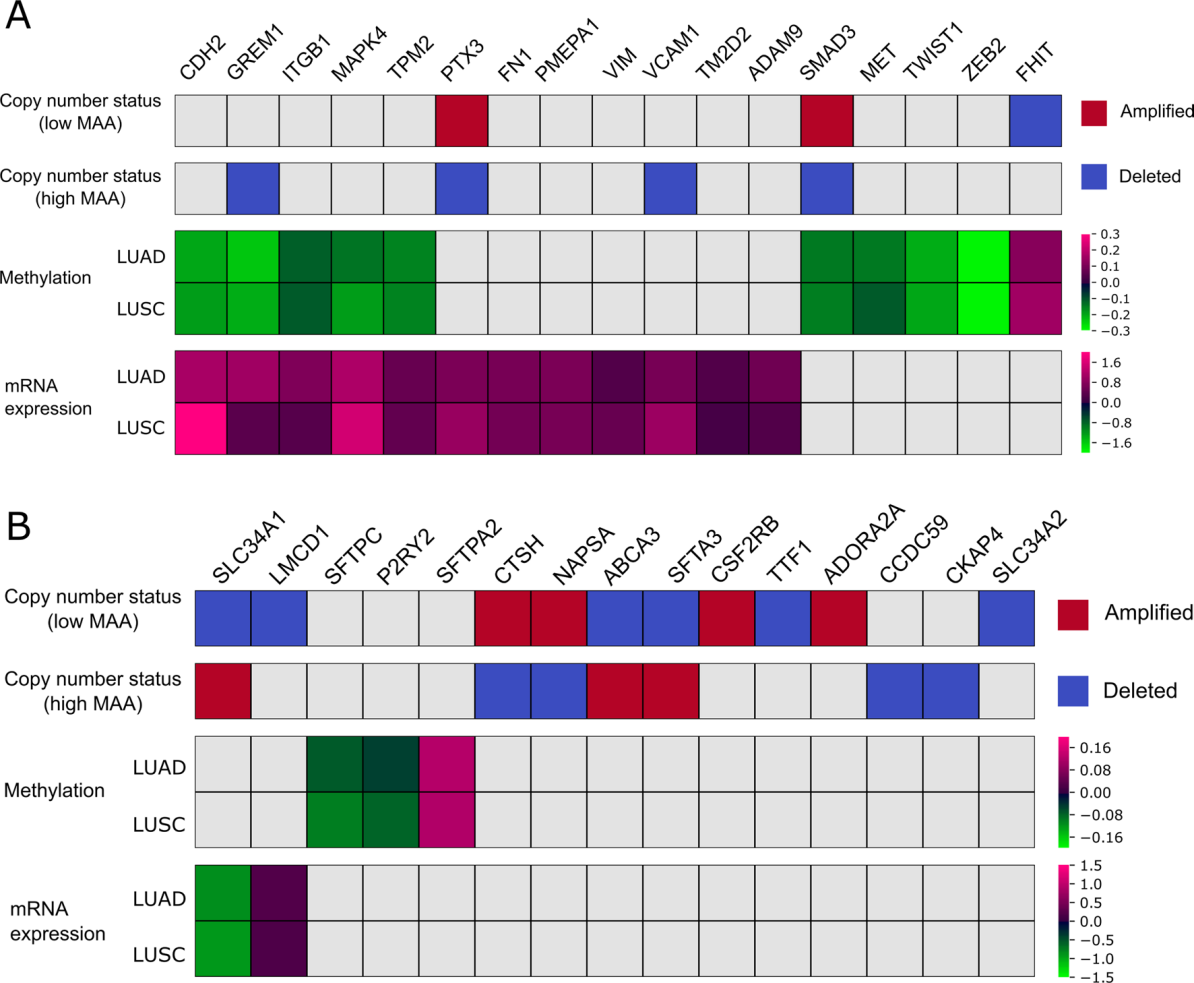
Multi-omics profiling of EMT-related genes in samples with MAA above and below the optimal separation threshold. The copy number (CN) status of the highlighted genes is shown in the first and second parts of the plot. The first line represents the CN status in the above threshold group and the second the CN status in the below threshold group. Red corresponds to genes found significantly amplified in the considered group, blue to deleted genes. The third part of the figure shows a heatmap that illustrates the results of the differential methylation analysis. The first line represents the logarithm of the fold change obtained in the comparison between LUAD samples with low MAA and LUAD samples with high MAA, while the second line shows the results of the same comparison carried out on LUSC samples. The last part of the plot portrays the results of differential expression analysis. The first and the second lines show, for LUAD and LUSC subtypes respectively, the logarithm of the fold change obtained in the comparison between low MAA samples and high MAA samples. Genes that are not significantly altered are depicted in grey.

We employed multi-omics data available for the TCGA dataset to characterize EMT-related genes at different MAA levels. Differential analyses on methylation and mRNA expression data were performed separately for LUAD and LUSC samples (see Material and Methods), comparing samples with low and high MAA. Differences in amplification and deletion frequency between low and high MAA samples were evaluated jointly for LUAD and LUSC samples. A summary of the most interesting altered genes is shown in Fig. 5. The action of a variety of genes known to promote EMT resulted enhanced in samples with low MAA. Some of these deregulations appeared at multiple profiling levels, such as the cadherin family member CDH2, which was both overexpressed and hypomethylated in low MAA samples, or FHIT, a tumor suppressor gene, which was both hypermethylated and deleted in low MAA samples.

### Low MAA samples are enriched in recurring lung cancer mutations

Mutation analysis, performed jointly on LUAD and LUSC samples, identified three statistically significant alterations. TP53 was more frequently altered in low MAA samples (low: 75.9%, high: 59.6%; *p* = 0.002), like RASA1 (low: 7.6%, high: 1.2%; *p* = 0.008). KRAS, the most frequent oncogene driver mutation in NSCLC^26^, had an increased mutation frequency in high MAA samples (low: 9.2%, high: 20.7%; *p* = 0.02). Furthermore, out of 14 samples (4 LUAD, 10 LUSC) presenting concurrent mutations of RASA1 and NF1, recently proposed as defining a novel NSCLC subtype transversal to LUAD and LUSC^27,28^, 12 belonged to the low MAA subset.

### Low MAA cell lines show increased sensitivity to the FDA-approved chemotherapy drug Vinorelbine

**Figure 6:**
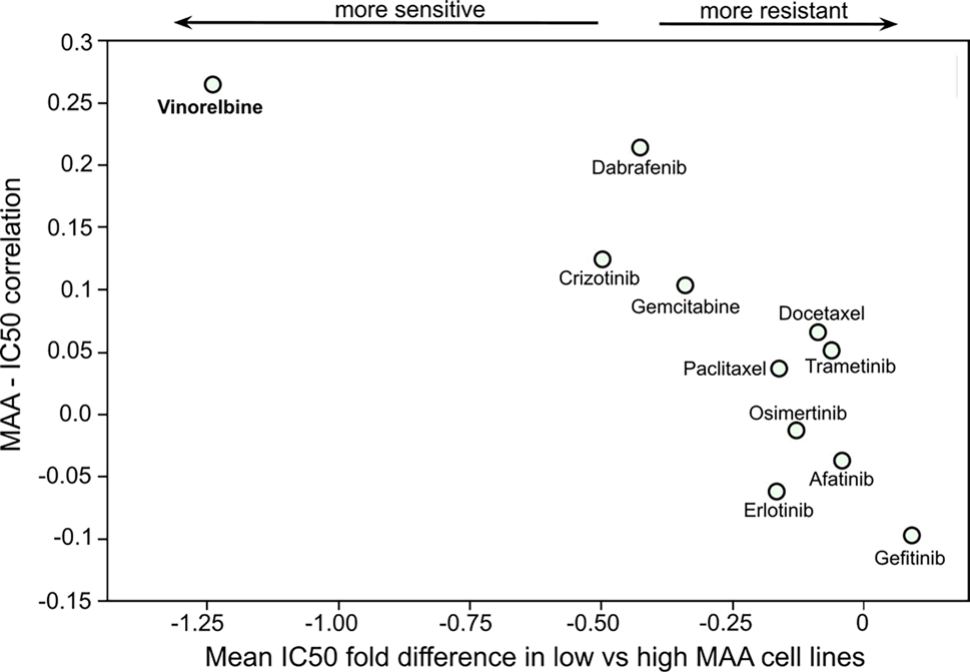
Relationship between MAA level and drug sensitivity in NSCLC cell lines for drugs approved by FDA for NSCLC treatment. Anti-mitotic Vinorelbine presents the largest change in sensitivity—higher in low MAA cell lines, compared to high MAA ones—paired with the highest correlation between IC50 and MAA value.

The association between MAA and drug sensitivity was assessed in NSCLC cell lines by comparing IC50 values of a selection of FDA-approved drugs for NSCLC treatment in a set of cell lines displaying different MAA levels. The anti-mitotic chemotherapy drug Vinorelbine showed the strongest variation in sensitivity between low and high MAA cells (FDR = 0.22, Fig. 6). Vinorelbine sensitivity values were also the most positively correlated (Pearson’s correlation) to MAA across cell lines.

## Discussion

The estimation of the two master regulators’ “activities” synthesized by the MAA index provides a powerful tool to understand the prognostic role of TTF1 and ΔNp63 through a more comprehensive view, beyond the mere assessment of their individual positivity. The MAA, computed by means of a dedicated systems biology approach, quantifies the strength of the mutual exclusivity between the two competing transcriptional programs in a more effective way than the simple comparison between the expression levels of the two genes (or protein products, when assessed by IHC).

Low MAA samples correspond to tumors where the “switch-like” behavior between the two transcription factors is disrupted towards a more balanced activity, hindering the realization of a specific gene expression program and likely limiting differentiation. Low MAA patients are characterized by worse prognosis; the shortening of survival times is in fact progressive, following the decrease of the MAA, as displayed by the Kaplan–Meier curves plotted for different MAA values.

Interestingly, the MAA prognostic value holds for the overall dataset, both for LUAD and LUSC. Despite the inherent high variability of transcriptome datasets, due to clinical and technological issues, results obtained on the TCGA cohort were confirmed in an independent group of patients, for both overall and recurrence-free survival, confirming the general validity of the proposed index. In addition, multivariate analysis indicated that the MAA was significantly associated with patients’ overall survival after adjustment for clinical variables as sex, age, smoking, and tumor stage. Univariate analysis, within each stage category, restated prognostic significance for all the stages except for stages IIIB and IV.

The annotation analysis performed to compare differentially expressed genes between low and high MAA samples showed the coherent enrichment of survival signatures specific for lung cancer, confirming further the prognostic significance of the MAA index.

The systematic analysis of gene set enrichment performed to compare low and high MAA tumors showed that upregulated genes in low MAA tumors are enriched for a multicancer invasiveness signature, along with EMT (Supplementary Fig. 1A) and collagen formation gene sets, suggesting a relevant contribution of extracellular matrix remodeling as expected in more aggressive cancer phenotypes.

Multi-omics profiling of EMT-related genes indicated undermethylation of several genes, accompanied by concurrent overexpression at transcript level for CDH2, GREM1, ITGB1, MAPK4 and TPM2. FHIT was the only gene that resulted overmethylated, coherently with its tumor suppressor function with roles in apoptosis and prevention of the EMT^29^. Conversely, key EMT master-regulators as TWIST1 and ZEB2 were found undermethylated.

With regards to genetic alterations, most clinical studies suggest that NSCLC with TP53 alterations have worse prognosis^30^ and RASA1 is a recognized strong driver of NSCLC and coherently, we found these genes more frequently altered in low MAA samples.

This does not happen for KRAS which is the most frequently mutated oncogene in NSCLC patients but it can be observed that it still remains an elusive target, from a therapeutic point of view^31^.

Furthermore, a negative enrichment of surfactant metabolism (Supplementary Fig. 1B) was found in low MAA samples; this can be, in principle, referred to a less differentiated phenotype but it finds interesting explanations in recent studies. Pocha et al.^32^ for example, showed that the high expression of surfactant pathway-related genes is a feature, shared by primary LUADs and LUAD brain metastases, that corresponds to an inflammatory and less immunosuppressive tumor environment correlating with prolonged survival. Lee et al.^33^ demonstrated, through experiments in vitro and in vivo, that downregulation of surfactant protein B (SP-B) is involved in the radiation-induced metastatic conversion of NSCLC and provided evidence that SP-B acts as a suppressor of NSCLC progression***.***

Also, the gene whose expression is most correlated to the MAA index was found to be the circadian gene hepatic leukemia factor (*HLF*), a bZIP transcription factor member of the proline and acidic-rich (PAR) protein family. Interestingly, in a very recent paper^38^ *HLF* was proposed as a prognostic biomarker in NSCLC, as in this context its downregulation predicts early relapse and distant metastasis. Mechanistically, its downregulation was shown to promote anaerobic metabolism to support anchorage-independent growth of NSCLC cells under low nutritional condition by activating *NF-κB*/p65 signaling through disruption of PPAR translocation^38^. Conversely, its upregulation inhibits lung colonization and metastasis, thus deserving attention as a novel actionable target in NSCLC.

In conclusion, the MAA index can represent a valuable prognostic in NSCLC, providing a unifying description beyond different subtypes and possible insights about mechanistic aspects.

## Methods

### Gene expression data

The analyses were performed on publicly available data on NSCLC from TCGA. Level 3 RNA-Seq gene isoforms expression data were retrieved from *FireBrowse* website (http://firebrowse.org/) for 517 LUAD and 501 LUSC, along with 110 control samples. Scaled estimates data were used (*illuminahiseq_rnaseqv2-RSEM_isoforms (MD5)*).

To confirm results obtained on the main dataset, survival analyses were replicated on a second dataset retrieved from the public repository NCBI Gene Expression Omnibus (series GSE41271). This dataset included gene expression data from microarray profiling for 275 lung cancer samples, from which we kept those relevant to our study: 183 LUAD, 80 LUSC and 2 lung adenosquamous carcinomas. In the case of genes with multiple transcripts, the instance with the highest variance across samples was kept.

### Reconstruction of TTF1 and p40 transcriptional networks

To evaluate the extent of influence of the two transcription factors (TFs) of interest, TTF1 and p40, we estimated their correlation with other genes across all the 1128 samples: statistical dependencies were computed in terms of mutual information by means of the Algorithm for the Reconstruction of Accurate Cellular Networks (ARACNe)^34^. For both TFs, the respective list of correlated genes (‘regulon’) was obtained as the union of all the transcripts correlated to their single isoforms. The isoforms employed at this step, as well as for the subsequent inference of activity, were those available from the TCGA dataset for the two regulators: ΔNp63α (uc003fsc.2), ΔNp63β (uc003fsd.2), ΔNp63γ (uc003fsb.2) and ΔNp63ε (uc010hzd.1) for p40; uc001wtt.2, uc001wtu.2 and uc001wtv.2 for TTF1. With the intention of obtaining strongly reliable regulons and keeping their size adequate for activity estimation, we set an upper bound of 10^–130^ for statistical significance.

### Master regulator activity analysis

The activity of the two master regulators in each sample was estimated by using the Virtual Inference of Protein-activity by Enriched Regulon analysis (VIPER) algorithm^35^. It estimates the strength of a transcription factor’s influence in a sample by evaluating the expression levels of its potential targets.

The activity levels of p40 and TTF1 were estimated using the expression of the genes included in the regulons previously obtained. For each TF, we considered their expression values as the sum of the expression levels of the single isoforms. Activity values were then used to calculate the mean absolute activity (MAA) index.

High values of MAA indicate that one regulator is strongly prevailing on the other and is driving the transcriptional regulation in the tumor. Conversely, low MAA values denote a balance in the activity between the two TFs.

### Survival analysis

Survival analyses were performed on both datasets, evaluating overall survival (OS) and recurrence-free survival (RFS). For the TCGA dataset, clinical data were downloaded from *cBioPortal*^36^ website. Out of 1016 patients with at least one tumor sample, 1003 presented information on survival status and 807 on recurrence status. Two patients having two tumor samples each were excluded from these analyses. For the second dataset, all but one sample had information on survival and recurrence status. Analyses were performed in R through the *survival* package. Statistical significance for Kaplan–Meier curves comparison were obtained via log-rank test. The optimal threshold for MAA index, maximizing the difference in terms of OS between the samples above and below such threshold, was found as the optimal cut point of the Cox regression model fit on the MAA distribution.

Cox proportional hazards models were applied in univariate and multivariate survival analysis adjusting for potential confounding factors. The p-values lower than 0.05 were deemed significant.

### Functional enrichment analysis

Gene expression data (RNA-Seq) were retrieved from *FireBrowse* website for a total of 1018 samples (LUAD, n = 517, LUSC, n = 501). We used DESeq2^37^ to compare raw counts between low and high MAA samples from LUAD and LUSC subtypes jointly, and performed a gene set enrichment analysis (GSEA) on the resulting gene ranking.

### Multi-omics data analysis

Additional omics data were obtained from the TCGA dataset to investigate the different molecular characteristics of samples with high and low MAA values. Methylation data measured by the Illumina Infinium HumanMethylation450 platform were available on *XenaBrowser*^38^ website for 826 samples (LUAD: 456, LUSC: 370). We performed a differential analysis comparing samples with MAA below the optimal separation threshold to samples with MAA above the same threshold. The analysis was carried out on gene expression data using DESeq2^37^, whereas a two-sided Welch t-test was applied to compare methylation levels in the two groups. The differential analyses were performed separately for LUAD and LUSC samples. We then selected genes statistically significant and showing the same behavior in both histotypes, e.g., significantly upregulated (or overmethylated) in low MAA group in both LUAD and LUSC subtypes.

We also retrieved copy number variations and somatic mutations data from *XenaBrowser* for 1010 (LUAD: 512, LUSC: 498) and 986 samples (LUAD: 509, LUSC: 477), respectively. Differences in mutation, amplification and deletion frequency in distinct MAA groups were tested using two-sided Fisher’s exact test, this time grouping LUAD and LUSC samples together.

*P*-values were corrected for multiple testing with the Benjamini–Hochberg procedure for all the analyses except for mutation enrichment, for which we employed the Bonferroni method. Adjusted *p*-values < 0.05 were deemed statistically significant.

### Drug sensitivity analysis

Drug sensitivity data were retrieved from The Genomics of Drug Sensitivity in Cancer (GDSC, second release) database^39^ to evaluate the association between MAA and sensitivity for 11 drugs that are approved for NSCLC treatment^40^. The dataset included IC50 values and paired RNA-seq data for 64 LUAD and 14 LUSC cell lines, identified among the available NSCLC cell lines according to their subtype (‘adenocarcinoma’ for LUAD, ‘squamous cell carcinoma’ for LUSC). Ln(IC50) data for the considered cell lines and drugs can be found in Supplementary Table S4. The MAA value for each sample was computed from basal gene expression data according to the same procedure described previously.

For each drug, correlation between IC50 and MAA across cell lines was measured through Pearson’s correlation coefficient, and mean IC50 fold change in low vs high MAA cell lines was computed.

### Ethical approval

This study was carried out on re-used publicly available data. No ethical approval was required in order to access the data.

### Supplementary Information

Supplementary Information.

## Data Availability

Publicly available datasets were analyzed in this study. The datasets supporting the conclusions of this article are available at the TCGA repository (LUAD and LUSC), and NCBI Gene Expression Omnibus (GSE41271).
