# Supplementary material for: Lack of imbalance between the master regulators TTF1/NKX2-1 and ΔNp63/p40 implies adverse prognosis in non-small cell lung cancer
Source: Sci Rep. 2024 Jan 30;14:2467. doi: 10.1038/s41598-024-52776-z (PMC10827720; doi:10.1038/s41598-024-52776-z)
Supplement: Supplementary file 1 — Supplementary Information. [file 41598_2024_52776_MOESM1_ESM.pdf]

LACK OF IMBALANCE BETWEEN THE MASTER REGULATORS *TTF1/NKX2-1* AND *ΔNp63/p40* IMPLIES ADVERSE PROGNOSIS IN NON-SMALL CELL LUNG CANCER

Martina Vescio [a, ^, §], Matteo Bulloni [a, §], Giuseppe Pelosi [c, d, #], Linda Pattini [a, b, #] *

[a] Department of Electronics, Information and Bioengineering, Politecnico di Milano, Milan, Italy

[b] CardioTech, IRCCS Centro Cardiologico Monzino, Milan, Italy

[c] Department of Oncology and Hemato-Oncology, University of Milan, Milan

[d] Inter-Hospital Pathology Division, IRCCS MultiMedica, Milan, Italy

* Correspondence to:

Linda Pattini, Department of Electronics, Information and Bioengineering, Politecnico di Milano, Piazza Leonardo da Vinci 32, 20133 Milan, Italy. Tel: +39 02 2399 3318; e-mail: linda.pattini@polimi.it.

[§] Both authors contributed equally as co-first authors.

[#] Both authors contributed equally as co-senior authors

^current affiliation: CardioTech, IRCCS Centro Cardiologico Monzino, Milan, Italy

Supplementary Figure S1

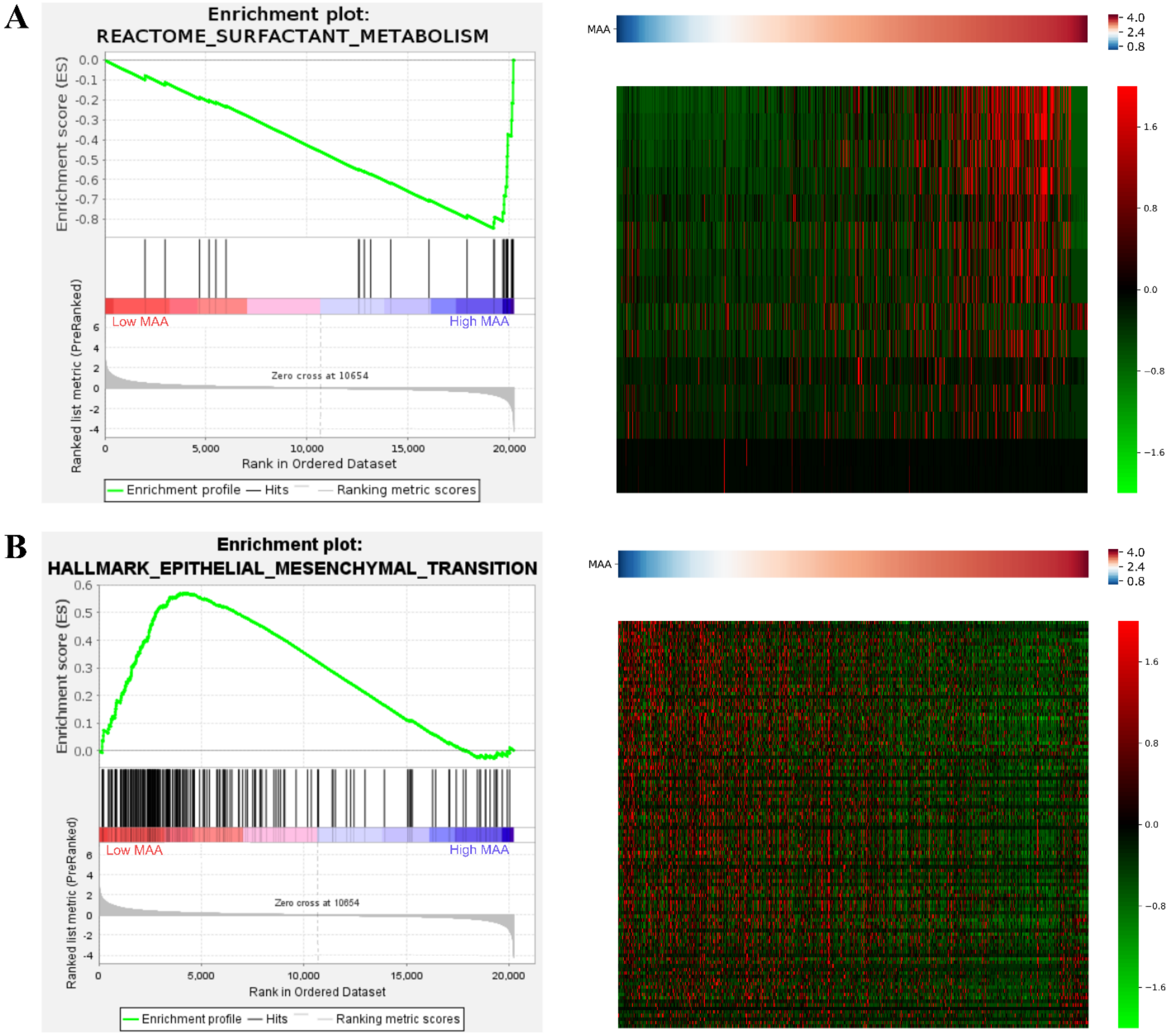

Supplementary Figure S1 Results of gene set enrichment analysis for Hallmark Epithelial Mesenchymal transition and for Reactome Surfactant Metabolism comparing low and high MAA tumors. Low MAA tumors are enriched for Hallmark Epithelial Mesenchymal transition (A), high MAA tumors are enriched for Reactome Surfactant Metabolism (B).

Suppl. Table S1 - Patient clinical characteristics_TCGA(LUAD LUSC)

Characteristic	n (%) LUAD	n (%) LUSC
**Age**		
Median (range)	66 (38-88)	68 (39-90)
**Sex**		
Female	277 (53.8)	130 (25.9)
Male	238 (46.2)	371 (74.1)
**Primary Tumor Site**		
Bronchial	0 (0.0)	10 (2.0)
L-Lower	78 (15.1)	76 (15.2)
L-Upper	123 (23.9)	137 (27.3)
R-Lower	97 (18.8)	108 (21.6)
R-Middle	21 (4.1)	18 (3.6)
R-Upper	181 (35.1)	133 (26.5)
Other	4 (0.8)	11 (2.2)
[Not Available]	11 (2.1)	8 (1.6)
**Location lung parenchyma**		
Central Lung	63 (12.2)	147 (29.3)
Peripheral Lung	127 (24.7)	93 (18.6)
[Not Available]	325 (63.1)	261 (52.1)
**Surgical Margin Resection Status**		
R0	344 (66.8)	398 (79.4)
R1	13 (2.5)	12 (2.4)
R2	4 (0.8)	4 (0.8)
[Not Available]	154 (29.9)	87 (17.4)
**Tumor Stage**		
T1	67 (13.0)	50 (10.0)
T1a	47 (9.1)	24 (4.8)
T1b	55 (10.7)	40 (8.0)
T2	168 (32.6)	172 (34.3)
T2a	82 (15.9)	87 (17.4)
T2b	27 (5.2)	34 (6.8)
T3	47 (9.1)	71 (14.2)
T4	19 (3.7)	23 (4.6)
[Not Available]	3 (0.6)	0 (0.0)
**Lymph Node Stage**		
N0	331 (64.3)	319 (63.7)
N1	96 (18.6)	131 (26.1)
N2	74 (14.4)	40 (8.0)
N3	2 (0.4)	5 (1.0)
[Not Available]	12 (2.3)	6 (1.2)
**Metastasis Stage**		
M0	346 (67.2)	411 (82.0)
M1	18 (3.5)	5 (1.0)

M1a	2 (0.4)	1 (0.2)
M1b	5 (1.0)	1 (0.2)
[Not Available]	144 (28.0)	83 (16.6)
**Neoplasm Disease Stage**		
Stage I	5 (1.0)	3 (0.6)
Stage IA	131 (25.4)	90 (18.0)
Stage IB	139 (27.0)	151 (30.1)
Stage II	1 (0.2)	3 (0.6)
Stage IIA	50 (9.7)	65 (13.0)
Stage IIB	71 (13.8)	94 (18.8)
Stage III	0 (0.0)	3 (0.6)
Stage IIIA	73 (14.2)	63 (12.6)
Stage IIIB	11 (2.1)	18 (3.6)
Stage IV	26 (5.0)	7 (1.4)
[Not Available]	8 (1.6)	4 (0.8)
**Patient Smoking History Category**		
1	75 (14.6)	18 (3.6)
2	119 (23.1)	133 (26.5)
3	135 (26.2)	83 (16.6)
4	168 (32.6)	250 (49.9)
5	4 (0.8)	5 (1.0)
[Not Available]	14 (2.7)	12 (2.4)

Suppl. Table S2 - Patient clinical characteristics_GSE41271

Characteristic	n (%)
**Patient stage**	
IA	47 (17.74)
IB	84 (31.70)
IIA	10 (3.77)
IIB	36 (13.58)
IIIA	48 (18.11)
IIIB	34 (12.83)
IV	5 (1.89)
IA Vs IIIB	1 (0.377)
**Sex**	
Male	144 (54.34)
Female	121 (45.66)
**Histology**	
Adenocarcinoma	183 (69.06)
Squamous cell carcinoma	80 (30.19)
Adenosquamous	2 (0.76)
**Race**	
Caucasian	235 (88.68)
African American	15 (5.66)
Hispanic	8 (3.02)
Asian	7 (2.64)
**Tobacco history**	
Yes	235 (89.69)
No	27 (10.31)
**Age**	
median (range)	64 (30 - 85)

Suppl. Table S3 – enrichment analysis: low vs high MAA samples.

Molecular Signature	SIZE	NES	NOM p-val	FDR q-val
ROSTY_CERVICAL_CANCER_PROLIFERATION_CLUSTER	132	2,479527	0	0
SHEDDEN_LUNG_CANCER_POOR_SURVIVAL_A6	420	2,407359	0	0
SOTIRIOU_BREAST_CANCER_GRADE_1_VS_3_UP	142	2,276754	0	0
NAKAYAMA_SOFT_TISSUE_TUMORS_PCA2_UP	86	2,276375	0	0
KONG_E2F3_TARGETS	94	2,275402	0	0
ZHAN_MULTIPLE_MYELOMA_PR_UP	43	2,233444	0	0
CROONQUIST_IL6_DEPRIVATION_DN	92	2,229023	0	0
KANG_DOXORUBICIN_RESISTANCE_UP	50	2,226927	0	0
FLORIO_NEOCORTEX_BASAL_RADIAL_GLIA_DN	174	2,15901	0	0
ZHOU_CELL_CYCLE_GENES_IN_IR_RESPONSE_24HR	113	2,143334	0	0
AUNG_GASTRIC_CANCER	51	2,13362	0	0
CROONQUIST_NRAS_SIGNALING_DN	68	2,128254	0	0
RICKMAN_HEAD_AND_NECK_CANCER_A	94	2,125769	0	0
REACTOME_ASSEMBLY_OF_COLLAGEN_FIBRILS_AND_OTHER_MULTIMERIC_STRUCTURES	58	2,114686	0	2,97E-05
KOBAYASHI_EGFR_SIGNALING_24HR_DN	236	2,110324	0	2,89E-05
CHIANG_LIVER_CANCER_SUBCLASS_PROLIFERATION_UP	165	2,103574	0	8,44E-05
ODONNELL_TFRC_TARGETS_DN	122	2,091932	0	7,81E-05
SARRIO_EPITHELIAL_MESENCHYMAL_TRANSITION_UP	171	2,076715	0	0,000149
SENGUPTA_NASOPHARYNGEAL_CARCINOMA_UP	277	2,066755	0	0,00019
FRASOR_RESPONSE_TO_SERM_OR_FULVESTRANT_DN	47	2,063688	0	0,000181
WINNEPENNINCKX_MELANOMA_METASTASIS_UP	149	2,054756	0	0,000239
ANASTASSIOU_MULTICANCER_INVASIVENESS_SIGNATURE	63	2,053685	0	0,000255
CROMER_TUMORIGENESIS_UP	56	2,05167	0	0,000292
BASAKI_YBX1_TARGETS_UP	259	2,046238	0	0,000348
GRAHAM_NORMAL_QUIESCENT_VS_NORMAL_DIVIDING_DN	86	2,042437	0	0,000375
FARMER_BREAST_CANCER_CLUSTER_2	32	2,025531	0	0,000532
RUIZ_TNC_TARGETS_DN	135	2,020852	0	0,00054
WU_APOPTOSIS_BY_CDKN1A_VIA_TP53	48	2,016527	0	0,000624
HALLMARK_EPITHELIAL_MESENCHYMAL_TRANSITION	194	2,013796	0	0,000686
WHITEFORD_PEDIATRIC_CANCER_MARKERS	112	2,004775	0	0,000903
HALLMARK_G2M_CHECKPOINT	186	1,988161	0	0,001242
BURTON_ADIPOGENESIS_3	100	1,986757	0	0,001254
DUTERTRE_ESTRADIOL_RESPONSE_24HR_UP	300	1,981425	0	0,001472
ZHOU_CELL_CYCLE_GENES_IN_IR_RESPONSE_6HR	77	1,98139	0	0,001452
CHARAFE_BREAST_CANCER_LUMINAL_VS_MESENCHYMAL_DN	429	1,979735	0	0,001432
CHICAS_RB1_TARGETS_GROWING	224	1,972566	0	0,00161
DOANE_BREAST_CANCER_ESR1_DN	45	1,969753	0	0,001664
HALLMARK_E2F_TARGETS	189	1,969327	0	0,001669
MORI_IMMATURE_B_LYMPHOCYTE_DN	88	1,968888	0	0,00164
RHODES_UNDIFFERENTIATED_CANCER	65	1,965008	0	0,001803
FUJII_YBX1_TARGETS_DN	185	1,962939	0	0,001814

KAUFFMANN_MELANOMA_RELAPSE_UP	57	1,961195	0	0,001867
ISHIDA_E2F_TARGETS	51	1,960371	0	0,001882
REACTOME_COLLAGEN_FORMATION	81	1,953523	0	0,00238
ODONNELL_TARGETS_OF_MYC_AND_TFRC_DN	43	1,950334	0	0,002591
BENPORATH_PROLIFERATION	133	1,950018	0	0,002573
EGUCHI_CELL_CYCLE_RB1_TARGETS	23	1,946811	0	0,002747
REACTOME_RESOLUTION_OF_SISTER_CHROMATID_COHESION	115	1,943162	0	0,002891
REACTOME_MET_PROMOTES_CELL_MOTILITY	41	1,940641	0	0,003049
FISCHER_G2_M_CELL_CYCLE	219	1,929301	0	0,003785
SHEPARD_BMYB_TARGETS	71	1,926231	0	0,00393
MISSIAGLIA_REGULATED_BY_METHYLATION_DN	111	1,921938	0	0,004143
LEE_EARLY_T_LYMPHOCYTE_UP	94	1,91625	0	0,004607
MOLENAAR_TARGETS_OF_CCND1_AND_CDK4_DN	47	1,915003	0	0,004703
HORIUCHI_WTAP_TARGETS_DN	292	1,911803	0	0,004915
WEST_ADRENOCORTICAL_TUMOR_MARKERS_UP	22	1,910951	0	0,004875
GAVIN_FOXP3_TARGETS_CLUSTER_P6	87	1,907215	0	0,005259
WHITFIELD_CELL_CYCLE_LITERATURE	42	1,906372	0	0,005231
CLASPER_LYMPHATIC_VESSELS_DURING_METASTASIS_DN	35	1,902631	0	0,005635
REACTOME_MET_ACTIVATES_PTK2_SIGNALING	30	1,896522	0	0,006232
KAMMINGA_EZH2_TARGETS	41	1,887977	0	0,00707
VECCHI_GASTRIC_CANCER_ADVANCED_VS_EARLY_UP	161	1,887028	0	0,007002
RORIE_TARGETS_OF_EWSR1_FLI1_FUSION_DN	27	1,878059	0	0,00813
REACTOME_TRANSCRIPTIONAL_REGULATION_OF_PLURIPOTENT_STEM_CELLS	31	1,87749	0	0,008124
MORI_LARGE_PRE_BII_LYMPHOCYTE_UP	81	1,876444	0	0,008239
PUJANA_BREAST_CANCER_WITH_BRCA1_MUTATED_UP	51	1,872892	0	0,008713
PID_SYNDECAN_1_PATHWAY	46	1,869194	0	0,009321
SHEDDEN_LUNG_CANCER_GOOD_SURVIVAL_A4	184	-2,77622	0	0
REACTOME_SURFACTANT_METABOLISM	28	-2,26264	0	0,000462

Complete list of significantly enriched gene sets (FDR q-value <0.01) in the comparison between low and high MAA samples.

Suppl. Table S4 – Drug sensitivity - Cell lines (GDSC repository, second release) and corresponding ln(IC50) for each drug considered.

Cell line Name	Model ID	COSMIC ID	Tissue sub-type	Vinorelbine_LN_IC50	Dabrafenib_LN_IC50	Crizotinib_LN_IC50	Gemcitabine_LN_IC50	Docetaxel_LN_IC50	Paclitaxel_LN_IC50	Trametinib_LN_IC50	Osimertinib_LN_IC50	Afatinib_LN_IC50	Erlotinib_LN_IC50	Gefitinib_LN_IC50
A549	SIDM00903	905949	lung_NSCLC_adenocarcinoma	-3,81144	3,573702	1,686823	-3,595637	-5,703386	-4,467824	-3,162699	1,212945	0,980552	2,117848	2,610555
ABC-1	SIDM00494	906791	lung_NSCLC_adenocarcinoma	-3,472322	4,42524	4,825354	-1,620566	-3,26112	-3,399604	2,890475	2,841505	3,11684	5,695293	3,264544
COR-L105	SIDM00513	906805	lung_NSCLC_adenocarcinoma	0,987477	4,856988	3,557925	-0,09608	-1,99775	-2,0909	-1,974958	-1,382719	-3,12962	-0,482667	-0,816615
Calu-3	SIDM00922	687777	lung_NSCLC_adenocarcinoma	1,79825	6,553622	2,972961	0,144707	-1,072924	-0,147948	-0,041582	0,105057	-1,763451	3,134475	2,949563
Calu-6	SIDM00921	724859	lung_NSCLC_adenocarcinoma	-4,315537	3,424857	3,365211	1,054217	-5,139113	-4,147054	-3,396511	3,48159	2,265253	3,90273	4,44232
EBC-1	SIDM00486	753554	lung_NSCLC_squamous_cell_carcinoma	-2,47851	4,655137	-2,855823	-1,962287	-3,765858	-3,344095	-3,941945	1,171696	2,695661	2,914487	4,12211
EKVX	SIDM00119	905970	lung_NSCLC_adenocarcinoma	0,375648	5,865378	6,839838	3,899284	0,052043	-0,094116	-0,700379	1,164936	0,347996	1,725803	2,743913
EMC-BAC-1	SIDM00048	1503369	lung_NSCLC_adenocarcinoma	-4,606351	4,171602	1,468441	-0,398163	-4,904473	-4,007609	-1,214814	-0,042516	-1,534185	1,684803	1,583742
EMC-BAC-2	SIDM00047	1503370	lung_NSCLC_adenocarcinoma	-4,886525	4,221772	2,288892	-2,833023	-4,824965	-2,820186	-1,019092	0,664314	1,27148	1,487932	2,450382
EPLC-272H	SIDM01044	753556	lung_NSCLC_squamous_cell_carcinoma	-2,169941	5,008004	2,326456	-0,714713	-5,206959	-4,350509	-1,53475	0,749272	0,229596	2,020633	1,430825
H3255	SIDM00046	1247873	lung_NSCLC_adenocarcinoma	-0,67818	4,734725	4,805109	2,133611	-2,93475	-1,945719	1,325322	-2,523354	-3,247328	0,325377	-0,437893
HARA	SIDM00598	1240142	lung_NSCLC_squamous_cell_carcinoma	-5,077678	5,356713	1,747515	-2,523683	-6,751161	-4,861483	-0,249039	0,781305	0,477308	1,796949	1,938616
HCC-15	SIDM01072	1240143	lung_NSCLC_squamous_cell_carcinoma	-3,97894	4,626194	2,211855	-1,693637	-6,299469	-5,140013	0,421306	2,527034	2,654064	2,156613	2,848972
HCC-44	SIDM01069	1240145	lung_NSCLC_adenocarcinoma	-4,383511	4,645323	1,434753	-1,196009	-5,483417	-4,28159	-2,292492	2,473396	1,428939	3,8079	3,867033
HCC-78	SIDM01068	1290908	lung_NSCLC_adenocarcinoma	-1,322364	6,179551	1,591165	-3,681265	-3,513913	-1,664591	0,79674	0,963438	1,13192	4,151055	4,021238
HCC-827	SIDM01067	1240146	lung_NSCLC_adenocarcinoma	-1,829383	6,478078	3,551241	2,490013	-3,041062	-0,622188	2,246463	-3,590724	-3,934364	-2,731313	-2,735744
HOP-62	SIDM00133	905972	lung_NSCLC_adenocarcinoma	-2,204631	5,967113	3,642028	-1,004775	-2,728032	-1,277833	-0,041027	2,742171	3,657268	2,850358	4,766858
KNS-62	SIDM00606	753569	lung_NSCLC_squamous_cell_carcinoma	-2,371205	5,99323	3,939425	0,172241	-4,168176	-2,862512	-1,483035	2,206487	2,35975	3,255774	3,199834
LC-1-sq	SIDM00300	1298223	lung_NSCLC_squamous_cell_carcinoma	-1,00099	5,727876	3,146198	0,875649	-3,340118	-3,546918	1,174232	1,236521	1,055102	0,904653	1,024316
LC-2-ad	SIDM00297	907786	lung_NSCLC_adenocarcinoma	-3,35709	3,858644	1,700236	-4,31711	-5,471516	-5,100196	0,008447	1,639399	1,615891	2,1297	3,632084
LK-2	SIDM00548	687787	lung_NSCLC_squamous_cell_carcinoma	-5,224651	5,336498	1,11201	-0,895313	-5,78375	-3,839013	0,534384	1,440981	2,062306	3,759009	4,260984
LOU-NH91	SIDM00341	1298226	lung_NSCLC_squamous_cell_carcinoma	-4,668312	4,026548	4,258764	-1,226761	-5,599346	-3,438652	1,504146	0,29367	0,324134	0,397651	1,416904
LXF-289	SIDM00339	753592	lung_NSCLC_adenocarcinoma	-2,330038	5,21264	1,604291	-0,420679	-5,798938	-5,270194	-0,179289	2,343446	1,903123	3,008299	3,800499
NCI-H1355	SIDM00645	724866	lung_NSCLC_adenocarcinoma	-2,682911	6,265707	3,302522	0,64634	-3,999984	-0,752135	1,043015	3,228194	2,82274	3,164464	4,88491
NCI-H1435	SIDM00658	1298347	lung_NSCLC_adenocarcinoma	0,433572	3,218569	3,522454	2,45928	-2,719733	-1,8457	1,762133	2,21542	1,531175	3,074526	3,205247
NCI-H1437	SIDM00734	687794	lung_NSCLC_adenocarcinoma	-5,077501	4,77569	2,20107	-0,712924	-6,55891	-5,171533	-3,163633	2,466979	2,94428	3,568377	2,271589
NCI-H1563	SIDM00751	753600	lung_NSCLC_adenocarcinoma	1,132442	5,688328	3,277154	4,497488	-2,829781	-0,998775	2,057999	0,882932	1,501893	2,142612	2,896238
NCI-H1568	SIDM00750	1298348	lung_NSCLC_adenocarcinoma	0,118136	5,859393	4,175511	1,713275	-1,864158	0,317522	0,313144	0,469193	0,401117	0,05517	0,839573
NCI-H1573	SIDM00749	908472	lung_NSCLC_adenocarcinoma	-0,537831	4,931875	4,14048	5,490125	-3,541195	-1,022196	-0,27161	3,1689	2,059557	1,257048	2,963668
NCI-H1623	SIDM00747	687798	lung_NSCLC_adenocarcinoma	-1,005024	5,451125	5,301596	-2,948538	-3,584264	-1,343722	-0,234914	0,196595	-1,746032	0,497119	1,494992
NCI-H1648	SIDM00746	687799	lung_NSCLC_adenocarcinoma	-4,093568	4,704491	2,284294	2,711229	-5,319793	-4,835589	-1,4398	-0,701592	-1,540761	-0,095164	0,283375
NCI-H1650	SIDM00745	687800	lung_NSCLC_adenocarcinoma	2,361168	6,937526	4,909036	2,772292	-1,993333	0,07064	3,750328	0,407872	1,798	1,153674	2,803578
NCI-H1651	SIDM00744	910900	lung_NSCLC_adenocarcinoma	-3,023171	4,769027	3,514468	-1,029471	-5,236064	-2,995784	2,248012	1,780943	2,771331	2,566453	3,047637
NCI-H1666	SIDM00743	908473	lung_NSCLC_adenocarcinoma	-1,525702	5,526574	4,053034	0,244266	-3,991203	-0,691604	-2,550487	0,447561	-0,503551	0,257436	0,574708
NCI-H1693	SIDM00742	687802	lung_NSCLC_adenocarcinoma	0,972189	6,657505	4,588866	2,04209	-0,824133	0,47104	0,797341	1,1113	0,520163	1,797712	2,785947
NCI-H1703	SIDM00740	908474	lung_NSCLC_adenocarcinoma	-5,888716	3,860093	2,523926	-4,587321	-5,701442	-4,64152	2,647127	1,834172	0,497171	3,38427	3,518444
NCI-H1734	SIDM00739	722058	lung_NSCLC_adenocarcinoma	-4,836305	4,117581	1,752875	-4,748423	-5,292858	-5,296132	-1,358652	1,873261	3,99056	3,508	3,300381
NCI-H1755	SIDM00738	908475	lung_NSCLC_adenocarcinoma	-3,298669	3,888041	1,462928	1,613197	-4,696667	-3,858454	-0,867392	1,890945	1,574516	2,892298	4,20082
NCI-H1781	SIDM00754	1298350	lung_NSCLC_adenocarcinoma	-1,175681	5,128731	4,005889	-3,459063	-3,863125	-2,579461	0,745938	-0,34841	-1,821887	1,889964	1,806178
NCI-H1792	SIDM00771	724868	lung_NSCLC_adenocarcinoma	-5,096235	4,241859	1,580265	-3,16223	-5,538592	-4,83372	-2,338602	2,19357	1,729079	2,419636	3,079159
NCI-H1793	SIDM00755	908463	lung_NSCLC_adenocarcinoma	-1,902237	7,104562	6,804677	0,475289	-2,666929	-0,313534	2,475268	3,702049	3,452984	3,94859	4,35016
NCI-H1838	SIDM00769	687807	lung_NSCLC_adenocarcinoma	2,196017	5,044014	4,669215	4,790409	-0,684154	0,138866	2,12001	3,462617	2,446471	3,86942	3,052172
NCI-H1869	SIDM00768	1240183	lung_NSCLC_squamous_cell_carcinoma	0,89023	5,279527	4,125445	3,804342	-2,483241	-1,624099	-0,12368	0,085523	1,362469	0,438622	1,040692
NCI-H1944	SIDM00762	1240185	lung_NSCLC_adenocarcinoma	-3,749241	5,107768	3,280145	-2,456923	-4,553183	-0,618837	-0,382753	1,088707	1,546245	1,731054	3,247688
NCI-H1975	SIDM00759	924244	lung_NSCLC_adenocarcinoma	-2,782	4,821605	3,409715	-0,979499	-5,090236	-3,22578	-0,830559	-3,272124	-1,13203	1,813576	2,613279
NCI-H1993	SIDM00758	908476	lung_NSCLC_adenocarcinoma	-3,972474	4,748584	-1,1056	-2,324953	-4,725927	-3,56449	-1,903022	2,091601	1,237528	3,366161	3,929136
NCI-H2009	SIDM00756	724873	lung_NSCLC_adenocarcinoma	-3,735816	5,80168	2,125645	0,146285	-5,529811	-3,573264	-1,943845	2,373773	2,002324	2,964772	3,556171
NCI-H2023	SIDM00753	1240187	lung_NSCLC_adenocarcinoma	-2,704877	4,631107	1,503841	-2,521227	-4,943944	-3,746398	0,429118	3,062089	1,813578	3,418802	4,166171
NCI-H2030	SIDM00715	722045	lung_NSCLC_adenocarcinoma	-2,502982	4,513668	2,233445	3,315708	-3,443224	-3,92312	-0,528728	0,675624	0,9838	1,939703	3,187489
NCI-H2085	SIDM00709	687812	lung_NSCLC_adenocarcinoma	-0,060483	5,35739	2,96634	2,885272	-3,805236	-2,617928	2,081873	1,773792	2,331593	4,261567	3,298336
NCI-H2087	SIDM00708	724834	lung_NSCLC_adenocarcinoma	-2,851245	3,743291	3,451522	-0,642437	-4,091086	-2,175006	-1,768785	3,411423	2,961433	4,345665	4,749029
NCI-H2122	SIDM00702	722046	lung_NSCLC_adenocarcinoma	-5,977987	4,332386	1,207042	-2,634238	-6,92488	-5,269815	-3,945743	0,751953	0,457411	0,527964	1,513941
NCI-H2170	SIDM00716	687815	lung_NSCLC_squamous_cell_carcinoma	-2,471744	3,416825	1,471723	-4,812863	-5,529146	-4,488856	3,914326	-2,2718	-3,043466	0,554039	0,812171
NCI-H2228	SIDM00729	687816	lung_NSCLC_adenocarcinoma	0,255178	5,53795	1,171946	-1,372782	-4,147335	-2,139384	-0,56695	3,072226	2,359901	3,596674	4,021651
NCI-H226	SIDM00139	905941	lung_NSCLC_squamous_cell_carcinoma	0,023935	6,095585	3,827803	3,334964	-2,000947	0,814182	3,260629	4,519852	4,726244	5,152934	4,832462
NCI-H2291	SIDM00728	724874	lung_NSCLC_adenocarcinoma	-0,476811	7,138808	4,013059	2,502293	-1,510957	-0,750638	-3,176646	1,810679	1,72675	4,158938	3,533
NCI-H23	SIDM00138	905942	lung_NSCLC_adenocarcinoma	-3,940107	5,275803	2,443789	-2,642506	-3,731635	-1,786905	0,35518	2,942427	2,135284	3,108025	3,371457
NCI-H2342	SIDM00727	687819	lung_NSCLC_adenocarcinoma	1,072866	6,135436	3,687204	2,815828	-1,029403	-0,722021	2,387544	2,64891	1,843759	3,677234	4,047942
NCI-H2347	SIDM00726	687820	lung_NSCLC_adenocarcinoma	2,860225	6,762222	6,284836	5,136898	0,835716	0,604207	0,231131	3,553966	4,069873	4,958129	5,799634
NCI-H2405	SIDM00724	687821	lung_NSCLC_adenocarcinoma	3,108321	4,086516	6,428392	1,619753	0,334997	1,119509	-2,278731	3,333193	6,318374	3,200485	4,78697
NCI-H292	SIDM00493	753604	lung_NSCLC_adenocarcinoma	-6,013829	5,081437	1,255218	-4,063171	-6,661584	-5,507217	-3,786103	-1,083086	-1,419728	0,319285	1,580946
NCI-H3122	SIDM00137	1240190	lung_NSCLC_adenocarcinoma	-4,162653	4,449157	-0,502036	-2,678715	-4,522562	-3,685984	-2,031478	2,151088	1,910556	4,279557	4,323347
NCI-H322M	SIDM00117	905967	lung_NSCLC_adenocarcinoma	-2,594962	3,046952	3,559695	-1,294323	-3,795316	-1,60769	-3,142186	-1,380772	-2,912927	-1,215501	-0,851457
NCI-H358	SIDM00718	908465	lung_NSCLC_adenocarcinoma	-1,851081	5,176917	2,436218	0,83309	-4,139482	-2,82989	-2,204166	0,645906	-0,548867	1,983462	2,943489
NCI-H441	SIDM00925	908460	lung_NSCLC_adenocarcinoma	2,315816	7,293837	4,117515	2,564545	-1,691504	-0,020625	2,113897	5,459908	2,054621	3,099466	4,925241
NCI-H520	SIDM01130	908443	lung_NSCLC_squamous_cell_carcinoma	-4,365314	5,58069	2,714463	-2,568888	-4,976831	-2,705048	2,547082	2,669163	1,679008	3,463069	3,975482
NCI-H522	SIDM00116	905944	lung_NSCLC_adenocarcinoma	-4,284544	4,157647	4,045613	0,282979	-4,635011	-3,398682	2,28866	1,852038	1,63302	2,835668	2,519677
NCI-H596	SIDM01127	908459	lung_NSCLC_adenocarcinoma	-0,936249	5,918583	3,810119	3,774334	-3,977612	-1,566744	1,968563	3,506167	2,124712	3,884746	3,288105
NCI-H650	SIDM01124	722066	lung_NSCLC_adenocarcinoma	-2,861497	5,094364	3,116963	1,068273	-2,43373	-1,601221	0,460317	2,874454	2,392672	3,446104	2,496927
NCI-H838	SIDM01150	910399	lung_NSCLC_adenocarcinoma	-4,165223	4,600889	2,084949	-1,852138	-5,957045	-5,424894	0,14695	1,636913	1,456443	3,796658	3,142846
PC-14	SIDM00237	753608	lung_NSCLC_adenocarcinoma	-4,385505	5,298639	1,96523	-1,300925	-5,16635	-4,324686	-0,296021	-3,304365	-3,530867	-2,165675	-1,397038
RERF-LC-KJ	SIDM00356	1298537	lung_NSCLC_adenocarcinoma	-2,87439	5,46347	3,182994	4,811159	-4,443108	-1,373821	1,812806	1,415262	2,043837	0,902932	1,803538
RERF-LC-MS	SIDM00355	910931	lung_NSCLC_adenocarcinoma	-4,545752	2,990507	0,326156	-3,452757	-5,716663	-5,497767	-2,019906	1,983289	3,03158	3,109003	3,572504
RERF-LC-Sq1	SIDM00354	1298538	lung_NSCLC_squamous_cell_carcinoma	-3,151839	5,805716	2,516429	-1,268636	-4,798948	-3,424875	-2,381318	-0,186079	-0,949764	0,495385	0,356881
SK-LU-1	SIDM01108	909721	lung_NSCLC_adenocarcinoma	-0,637766	5,536916	3,201085	1,786704	-2,652585	-1,658056	-0,436267	4,560097	4,526636	4,942308	5,112853
SK-MES-1	SIDM01103	909728	lung_NSCLC_squamous_cell_carcinoma	-3,963676	2,75834	3,028007	-0,710119	-4,020776	-0,865901	-1,170242	1,042218	0,324819	1,358889	2,091955
SW1573	SIDM01163	724878	lung_NSCLC_adenocarcinoma	-0,138981	6,571956	4,69358	0,575826	-1,496825	0,196102	3,5489	4,609979	4,022561	5,340609	5,471111
VMRC-LCD	SIDM00320	713869	lung_NSCLC_adenocarcinoma	-2,791917	5,969894	1,827362	3,051052	-4,632165	-3,832255	3,244148	2,788954	2,481304	4,4595	3,95763
